# Role of the P2 residue of human alpha 1-antitrypsin in determining target protease specificity

**DOI:** 10.1371/journal.pone.0185074

**Published:** 2017-09-18

**Authors:** Hye-Shin Chung, Ji-Sun Kim, Sang Mee Lee, Soon Jae Park

**Affiliations:** 1 Alteogen Inc., Yuseong-gu, Daejeon, South Korea; 2 Department of Biological Sciences and Biotechnology, Hannam University, Daejeon, South Korea; Russian Academy of Medical Sciences, RUSSIAN FEDERATION

## Abstract

Alpha 1-antitrypsin (A1AT) is a serine protease inhibitor that mainly inhibits neutrophil elastase in the lungs. A variant of A1AT at the P1 position with methionine 358 to arginine (A1AT-Pittsburgh) is a rapid inhibitor of thrombin with greatly diminished anti-elastase activity. The P2 residue (position 357) of A1AT-Pittsburgh has been shown to play an important role in interactions with thrombin and kallikrein, but the role of P2 residue in wild-type A1AT has largely been unraveled. Here, we investigated the effects of P2 proline substitutions in wild-type A1AT on interactions with porcine pancreatic elastase (PPE) and human neutrophil elastase (HNE). The mutant A1AT proteins (P357A, P357D, P357K, P357L, P357N, P357S, and P357W) were less efficient than the wild-type A1AT at inhibiting PPE and HNE. Among the mutants, P357D did not form a complex with PPE, whereas P357L, P357N, and P357W showed significantly reduced complex formation with PPE. Surprisingly, mass spectrometry analysis revealed that P357D had two cleavage sites after the P9 alanine and the P3 isoleucine residues. Our results indicate that the size and negative charge of the R group of the P2 residue influence the interaction with elastases. Specifically, the negative charge at the P2 residue is disfavored and the resulting conformational changes in the reactive center loop upon interaction with PPE lead to cleavage at new sites. Overall, the results of this study demonstrate a previously unknown role for P2 residue in determining inhibitory specificity of A1AT.

## Introduction

**Fig 1:**
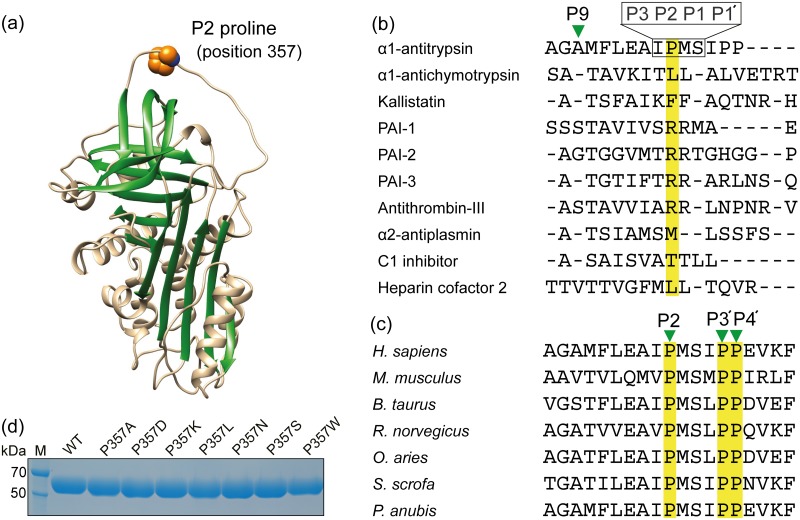
The structure of human A1AT, multiple sequence alignment, and the expression of recombinant A1AT proteins. (a) The location of P2 proline (position 357) in human A1AT (PDB ID: 3NE4). (b) Multiple sequence alignment of the reactive center loop sequences of serpin family proteins. (c) Multiple sequence alignment of the reactive center loop region of A1AT among different species. Multiple sequence alignments shown in (b) and (c) were conducted using ClustalW [18]. Nomenclature of Schechter and Berger was used for cleavage site positions of the protein substrate [4]. (d) SDS-polyacrylamide gel electrophoresis of the recombinant WT and mutant A1AT proteins after A1AT Select affinity chromatography. The proteins appeared on a SDS gel with a size of approximately 52 kDa.

Serine protease inhibitors (serpins) comprise a structurally similar superfamily of proteins that are distributed among animals, plants, viruses, and bacteria, and have evolved to regulate the activity of serine proteases, cysteine proteases, and metalloproteases [1]. Serpins are metastable in their native state, but undergo a conformational change with cognate proteases via a unique suicide cleavage mechanism [2, 3]. The reactive center loop, either fully exposed above the serpin molecule (Fig 1a, the structure of human α1-antitrypsin (A1AT)) or partially inserted into the central β-sheets, serves as bait to the proteases, which cleave the peptide bond at P1-P1′ residues (nomenclature of Schechter and Berger [4]), i.e., methionine 358 (P1)-serine 359 (P1′) for human A1AT (Fig 1b) [2, 3]. The resulting irreversible structural transition inserts the reactive center loop into two β-sheets of a serpin molecule, leading to the formation of a stable acyl-enzyme intermediate during catalysis by serine proteases [5, 6].

A serpin, human A1AT is a glycoprotein of 394 amino acids that mainly inhibits neutrophil elastase in the lungs [7]. A1AT is the most abundant serpin in the human body, with a normal serum concentration of 1.5–3.5 g/L [8]. The reactive center loop of A1AT, which consists of the canonical loop structure and β-strand conformation (residues 344–368), is highly flexible and dynamic in the native conformation (Fig 1a) [9]. Serpin specificity to proteases is determined by the amino acid sequence of the active center [10], which exhibits completely different amino acid sequences of the P3–P3′ segments among the serpin family proteins (Fig 1b) [11]. In particular, the P1 residue at the scissile bond is the critical determinant [12]. A natural variant of human A1AT at the P1 position with methionine 358 to arginine (also called A1AT-Pittsburgh) that causes bleeding disorders showed loss of inhibitory activity against elastases [13], but was found to inhibit thrombin [13], factor XIa, kallikrein, factor XIIf [14], and activated protein C [15]. The P2 proline (position 357) mutation of A1AT-Pittsburgh to alanine (alanine 357 and arginine 358) was shown to increase the rate of interaction with kallikrein relative to that observed with thrombin [16], but the mutation of P2 proline to glycine in A1AT-Pittsburgh decreased the association rate constant with thrombin [17]. However, the role of P2 residue in wild-type (WT) A1AT for determining serpin specificity to target proteases is largely unknown.

In this study, we investigated the effects of P2 proline substitutions in human WT A1AT on the interaction with porcine pancreatic elastase (PPE) and human neutrophil elastase (HNE). The P2, P3′, and P4′ proline residue of A1AT is highly conserved among different species (Fig 1c), despite its variance at the position within serpin family proteins (Fig 1b). The amino acids for P2 substitution included alanine and leucine (aliphatic), aspartate (acidic), lysine (basic), asparagine and serine (polar), and tryptophan (aromatic), representing each group of amino acids based on the chemical characteristics of the R groups. Here, we show for the first time that mutation of P2 proline to aspartate in human A1AT causes conformational changes in the reactive center loop upon its interaction with PPE that lead to subsequent cleavages after the carboxyl group of either the P9 alanine or the P3 isoleucine in A1AT. A previously unknown role for P2 residue in determining inhibitory specificity of A1AT to elastases has now been established.

## Materials and methods

### Materials

Human A1AT cDNA (clone hMU001448) was obtained from the Korea Research Institute of Bioscience and Biotechnology (Daejeon, South Korea). Porcine pancreatic elastase and HNE were purchased from Merck (Darmstadt, Germany). The synthetic fluorescent substrates, *N*-succinyl-alanine-alanine-alanine-*p*-nitroanilide for PPE and *N*-methoxysuccinyl-alanine-alanine-proline-valine-*p*-nitroanilide for HNE, were purchased from Sigma (St. Louis, MO, USA). Q-Sepharose FF column and an A1AT Select column were purchased from GE Healthcare (Piscataway, NJ, USA). All other reagents were obtained from Sigma, unless otherwise noted.

### Sequence analysis and structural model

Multiple sequence alignments were performed using ClustalW [18]. The structural model of the P357D mutant and PPE complex was generated using the Chimera software based on the crystal structure of A1AT-PPE (PDB ID: 2D26) [19].

### Site-directed mutagenesis

The human A1AT cDNA was prepared as previously described [20]. The A1AT gene, amplified by polymerase chain reaction, was subcloned into the *Xho* I and *Not* I sites of the mammalian expression vector pAV1. The cDNAs for the seven proline mutants were generated using an EZchange site-directed mutagenesis kit (Enzynomics, Daejeon, Korea) according to the manufacturer’s instructions. The primers used for site-directed mutagenesis were as follows:

P357A 5′-GCCatgtctatcccccccgaggtcaagttc-3′ and 5′-tatggcctctaaaaacatggcccc-3′
P357D 5′-GACatgtctatcccccccgaggtcaagttc-3′ and 5′-tatggcctctaaaaacatggcccc-3′
P357K 5′-AAAatgtctatcccccccgaggtcaagttc-3′ and 5′-tatggcctctaaaaacatggcccc-3′
P357L 5′-CTCatgtctatcccccccgaggtcaagttc-3′ and 5′-tatggcctctaaaaacatggcccc-3′
P357N 5′-ccatgtttttagaggccataAACatgtctatccccccc-3′ and 5′-gggggggatagacatgtttatggcctctaaaaacatgg-3′
P357S 5′-TCCatgtctatcccccccgaggtcaagttc-3′ and 5′-tatggcctctaaaaacatggcccc-3′
P357W 5′-TGGatgtctatcccccccgaggtcaagttc-3′ and 5′-tatggcctctaaaaacatggcccc-3′

The capital letters indicate the codon for the substituted amino acid. The mutations were confirmed by DNA sequencing.

### Expression and purification of A1AT proteins

The recombinant A1AT proteins were expressed and purified as previously described [20, 21]. Chinese hamster ovary (CHO)-K1 cells were grown in Isocove’s Modified Dulbecco’s Medium (IMDM) supplemented with 10% fetal bovine serum at 37°C in a 5% CO_2_ humidified incubator. CHO-K1 cells were transfected with each cDNA construct of WT or mutant A1AT using polyethyleneimine and grown in serum-free IMDM for an additional 96 h. The culture supernatant of transfected CHO-K1 cells was diluted with an equal volume of buffer A (20 mM sodium phosphate, pH 8.0), then applied to a Q-Sepharose FF column equilibrated with buffer A. After washing the column with buffer A, the proteins were eluted with a linear gradient of NaCl (70–400 mM) in buffer A. Next, the fractions containing A1AT were directly loaded onto an A1AT Select column equilibrated with buffer B (50 mM Tris·HCl, 150 mM NaCl, pH 7.5) and eluted with MgCl_2_ gradient (0–0.5 M) in buffer B. Fractions containing A1AT were concentrated using a Vivaspin 20 concentrator (Satorius) and dialyzed against phosphate-buffered saline. Purified proteins were analyzed for purity by sodium dodecyl sulfate (SDS)-polyacrylamide gel electrophoresis.

### Inhibition assays

Inhibitory activities of WT and mutant A1AT proteins were determined by incubation with PPE or HNE. Different concentrations of WT and mutant A1AT proteins (0, 5, 10, 15, 20, 30, 40, 50, and 60 nM) were prepared in assay buffer (20 mM Tris·HCl, 5 mM CaCl_2_, 0.01% Tween 80, pH 8.0). A1AT samples were mixed with 10 nM PPE or HNE, then incubated at 25°C for 30 min, after which the residual protease activity was measured using the synthetic fluorescent substrates, *N*-succinyl-alanine-alanine-alanine-*p*-nitroanilide for PPE or *N*-methoxysuccinyl-alanine-alanine-proline-valine-*p*-nitroanilide for HNE. Fluorescence was measured in 96-well plates at 405 nm for 5 min at 25°C using a SpectraMax M5 microplate reader (Molecular Devices).

### Determination of the association equilibrium constant (*K*_a_)

The *K*_a_ was determined by the equation [E]_f_ + [I]_f_ = [EI], where [E]_f_ and [I]_f_ are the residual enzyme and inhibitor with inhibitor complex, respectively, at equimolar concentrations of [E]_0_ and [I]_0_. The *K*_a_ values were calculated by linear regression of data plots of the concentrations of each WT and mutant A1AT protein over the residual elastase activities as previously described [22].

### A1AT-elastease complex formation assay

A1AT proteins were prepared by dilution in 20 mM Tris·HCl, 5 mM CaCl_2_, and 0.01% Tween 80 (pH 8.0). Mutant A1AT proteins were mixed with PPE at a specific molar ratio of A1AT to PPE (1/4, 1/2, 1/1, 2/1, and 4/1). Mixtures were incubated for 10 min at 25°C, then heated immediately with a reduced SDS gel-loading buffer (50 mM Tris·HCl, 2% SDS, 0.1% bromophenol blue, 10% glycerol, 100 mM DTT, pH 6.8) for 5 min at 95°C. Reaction products were separated on 10% SDS-polyacrylamide gels, visualized by Coomassie blue staining, then subjected to quantitative analysis with a CS Analyzer (Atto).

### Mass spectrometry analysis

The cleaved fragments of the P357D mutant hydrolyzed by PPE were analyzed in a Triple TOF 5600 Q-TOF LC/MS/MS system (AB Sciex) using an Ultimate 3000 RSLC high-performance liquid chromatography system (Thermo Fisher Scientific), including a degasser, an auto-sampler, a diode array detector and a binary pump. The liquid chromatography separation was performed on a Zorbax 300SB C8 column (30 nm, 5 μm, 2.1 × 50 mm,) with a mobile phase A (0.1% formic acid in water) and mobile phase B (0.1% formic acid in acetonitrile) applied at a flow rate of 0.25 mL/min under the conditions: the auto sampler was set at 4°C and the injection volume was 5 μL. Mass spectra were acquired under positive electrospray ionization with an ion spray voltage of 4500 V. The source temperature was 450°C, and the curtain gas, ion source gas 1, and ion source gas 2 were 35, 65, and 55 psi, respectively. Two full-scan mass spectra were acquired over an *m*/*z* range of 300–2000 in MS mode. The data were collected using the Analyst TF 1.7 software and analyzed using with PeakView 2.2. and ProteinPilot 4.5.

## Results

### Expression and purification of the P2 residue-substituted A1AT proteins

The plasmids for seven A1AT mutants (P357A, P357D, P357K, P357L, P357N, P357S, and P357W) were constructed using site-directed mutagenesis. Recombinant WT and mutant A1AT proteins were transiently expressed and secreted as soluble proteins in CHO-K1 cells. The recombinant proteins were purified by Q-Sepharose anion-exchange chromatography and A1AT Select affinity chromatography to homogeneity (>95% purity) as estimated by a CS analyzer 2.0 (data not shown). The recombinant WT and mutant A1AT proteins appeared on a SDS gel with a molecular weight of approximately 52 kDa (Fig 1d).

### Effects of P2 residue substitutions on the inhibitory activities of A1AT

**Fig 2:**
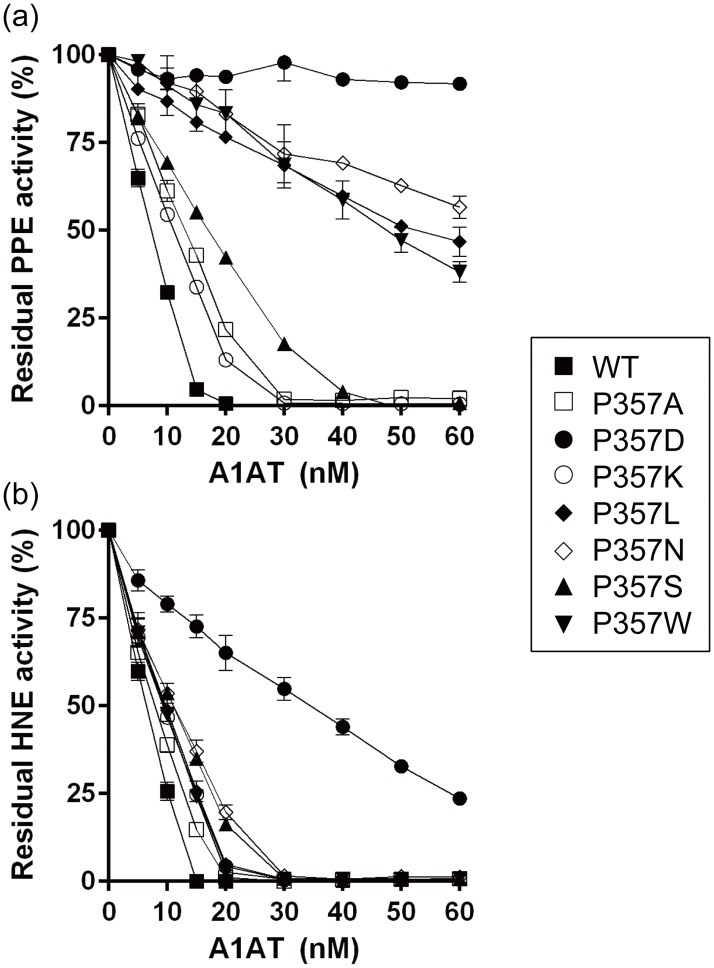
Protease inhibition assay of A1AT against PPE (a) and HNE (b). The residual activity of PPE and HNE is shown. Data correspond to the mean ± S.D. of three experiments.

The inhibitory activities of WT and seven A1AT mutants were evaluated against two elastases, PPE and HNE. Different concentrations (0–60 nM) of WT and mutant A1AT proteins were incubated with 10 nM of either PPE or HNE for 30 min at 25°C, after which the residual elastase activity was measured using synthetic fluorescent substrates. The degree of PPE inhibition was in the order of WT > P357K > P357A > P357S > P357L ≈ P357W > P357N > P357D (Fig 2a), indicating that proline is the preferred residue at the P2 position. The WT A1AT completely inhibited PPE activity at an A1AT concentration of 20 nM (Fig 2a). Interestingly, P357D showed no inhibition of PPE activity (Fig 2a). The P357L, P357N, and P357W mutants showed significantly reduced inhibitory activity towards PPE relative to WT and other mutants (Fig 2a).

On the other hand, the inhibitory effects of A1AT mutants on HNE activity were lower than those of the WT, but similar among the mutants (Fig 2b). These results indicate that the size and the negative charge of the R group of the P2 residue affects the interaction with elastases, having more distinct effects on PPE than HNE.

### Determination of association equilibrium constant (K_a_)

The *K*_a_ values of WT and mutant A1AT proteins to PPE and HNE were measured and are presented in Table 1. A1AT showed different binding affinity towards PPE and HNE. WT showed a *K*_a_ value of 1.1 × 10^10^ nM for PPE and 1.3 × 10^10^ nM for HNE. The binding affinity of WT and mutant A1AT proteins for PPE was in the order of WT > P357K > P357A > P357S > P357L > P357W > P357N, which are consistent with the order of PPE inhibition as shown in Fig 2a. Among the A1AT mutants, P357N showed a significantly lower binding affinity towards PPE with a reduced *K*_a_ value (9.1 × 10^7^ nM) approximately 120 fold lower than that of the WT (Table 1). P357W possessing a bulky hydrophobic indole group also exhibited substantially reduced binding affinity toward PPE (Table 1). Conversely, the binding affinity for HNE was in the order of WT > P357A > P357N > P357S > P357W > P357L > P357K > P357D (Table 1). The effects of P2 proline substitutions differed depending on the enzyme and were more evident with A1AT mutants with lower affinities. For example, lysine at position P2 was preferred for PPE, but not for HNE. Conversely, asparagine at position P2 was not favored for PPE, but was preferred for HNE. For both PPE and HNE, P357D showed either no binding towards PPE or the lowest binding affinity to HNE, supporting that aspartate is the most unfavorable substitution for P2 proline.

**Table 1:** Association equilibrium constants (*K*_a_) of WT and mutant A1AT proteins towards PPE and HNE. Data correspond to the means ± S.D. of three experiments.

A1AT	PPE	HNE
	*K*_a_ (nM)	*K*_a_ (nM)
WT	(1.1±0.1)×10^10^	(1.3 ± 0.3)×10^10^
P357A	(1.0± 0.1)×10^9^	(3.7 ± 0.3)×10^9^
P357D	N.D.^a^	(2.9 ± 0.6)×10^8^
P357K	(1.5± 0.1)×10^9^	(1.4 ± 0.2)×10^9^
P357L	(1.9±0.2)×10^8^	(1.5 ± 0.1)×10^9^
P357N	(9.1±1.3)×10^7^	(2.3 ± 0.2)×10^9^
P357S	(6.1± 0.2)×10^8^	(2.3 ± 0.1)×10^9^
P357W	(1.5±0.2)×10^8^	(2.1 ± 0.3)×10^9^

^a^N.D.: Non-Determined

### Complex formation of A1AT with PPE

**Fig 3:**
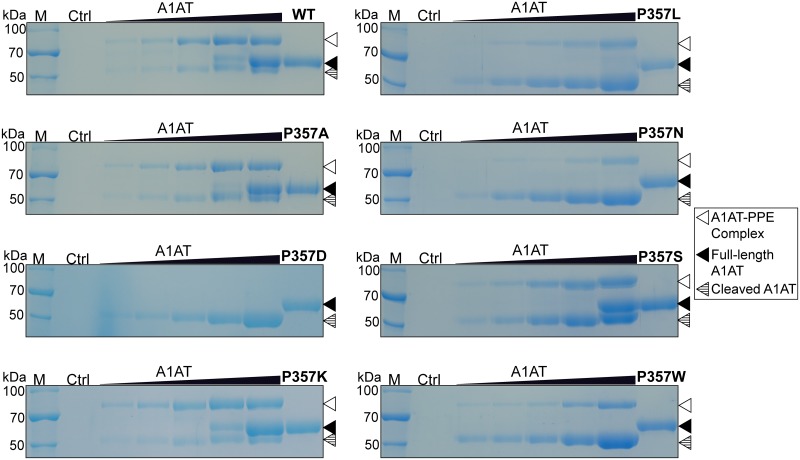
The complex formation of WT and mutant A1AT proteins with PPE. A1AT and PPE were incubated at molar ratios of 1/4, 1/2, 1/1, 2/1, and 4/1 (lanes 3 to 7 in each SDS gel). Lane 2: negative control (no protein). A representative 10% SDS gel is shown for WT and each A1AT mutant.

We next evaluated the complex formation of WT and mutant A1AT proteins with PPE in increasing molar ratios of A1AT to PPE (1/4, 1/2, 1/1, 2/1, and 4/1 ratios, respectively). The WT sample showed three protein bands on a SDS gel; SDS-resistant A1AT-PPE complex (approximately 70 kDa), full-length A1AT, and cleaved A1AT (Fig 3). The P357A, P357K, and P357S mutants showed three protein bands, similar to WT, suggesting that these mutations had little effect on complex formation with PPE. However, the P357L, P357N, and P357W mutants showed two proteins bands (A1AT-PPE complex and cleaved A1AT) upon interaction with PPE, although complex formation was retarded for the mutants, especially at the 4:1 ratio of A1AT to PPE with larger amounts of cleaved A1AT. These data are consistent with the significantly reduced inhibitory activity of the P357L, P357N, and P357W mutants (Fig 2a). Interestingly, P357D showed only one band (cleaved A1AT) and had no complex formation with A1AT (Fig 3). The absence of A1AT-PPE complex is consistent with the lack of inhibition of P357D against PPE as shown in Fig 2a. Although P357L, P357N, and P357W mutants showed cleaved fragments similar to P357D, they formed complexes with PPE on a SDS gel (Fig 3). With the exception of P357D, all mutants formed stable complexes with PPE in a A1AT concentration-dependent manner (Fig 3), but they required higher amounts of proteins for complex formation with PPE when compared with WT. For quantitative analysis, we measured the percentage of A1AT-PPE complex formation upon incubation of A1AT and PPE in a 2:1 molar ratio. Approximately 53% of WT A1AT formed complexes with PPE, followed by P357A (54%), P357K (44%), P357S (35%), P357L (21%), P357W (17%), and P357N (9%).

### Mass spectrometry analysis of the fragments of P357D

**Fig 4:**
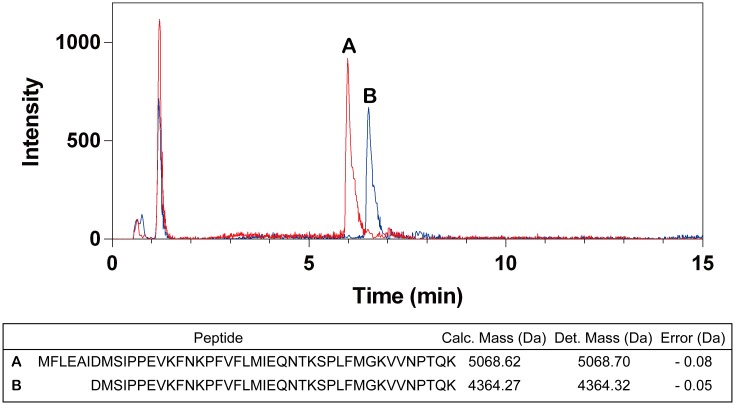
Mass spectrometry analysis of the cleaved fragments of P357D. Chromatogram and list of identified peptides. Peptides A and B were cleaved by PPE after the carboxyl group of the P9 alanine and the P3 isoleucine residues, respectively. Calculated peptide mass was compared with that of the observed mass.

To elucidate whether the cleavage site(s) of P357D is(are) the same as those of WT A1AT following treatment with P1 methionine, P357D was incubated with PPE for 30 min at 25°C, after which the mixture was subjected to mass spectrometry analysis. Surprisingly, two peptides with a molecular mass of 5068.70 Da and 4364.32 Da were identified (Fig 4). The molecular mass corresponds to a peptide consisting of the polypeptide with runs from methionine 351 to lysine 394 and another peptide consisting of the polypeptide with runs from aspartate 357 to lysine 394 (Fig 4). The data indicate that the P357D mutant had two different sites that could be cleaved by PPE, one after the carboxyl side of the P9 alanine and another after the carboxyl side of the P3 isoleucine.

## Discussion

A1AT mainly inhibits neutrophil elastase, and its specificity is determined by the amino acid residues in the reactive center loop, primarily the P1 residue [12, 13]. The amino acid residues adjacent the P1-P1′ scissile bond have also been shown to affect serpin specificity, but with less importance than the P1 residue [23]. Serpins are known to have exosites in addition to the P1 determinant for effective protease binding [23]. The P2 and P3′ proline residues of A1AT form hydrophobic head-to-head contacts with leucine 224, holding the region in the canonical conformation [9]. The mutation of P2 proline to alanine or glycine in A1AT-Pittsburgh changed the specificity to kallikrein and thrombin [16, 17]. The interactions between the P2 residue of a serpin and the S2 binding site of a protease have also been reported for other serpins, including α1-antichymotryspin [24] and kallistatin [25].

**Fig 5:**
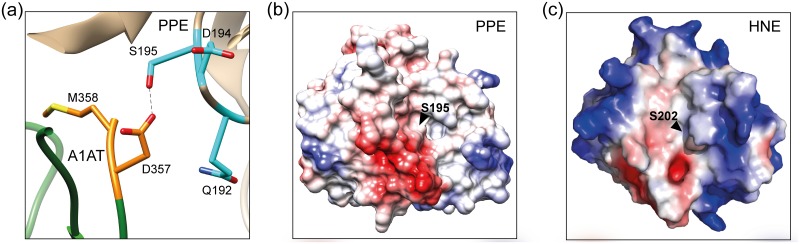
A1AT-PPE interaction and surface charge display of PPE and HNE. (a) Structural model of P357D and PPE based on the structure of A1AT-PPE (PDB ID: 2D26). Interaction between aspartate 357 of P357D and serine 195 of PPE is shown. (b) Surface charge display of PPE. (c) Surface charge display of HNE. Negative charges are shown in red and positive charges in blue.

X-ray crystallography revealed that the reactive center loop of A1AT has no ordered secondary structure and is instead an extended flexible structure [9]. The three conserved proline residues at the P2, P3′, and P4′ positions in the reactive center loop of A1AT (Fig 1c) may provide a firm and rigid loop structure and suggest there is a high affinity between A1AT and target proteases as the proline residue provides rigidity to the protein structure [9, 26]. The mutation of P2 proline to other amino acid residues disrupt the legitimate structure and may cause the reactive site area more to be flexible than that of WT. The crystal structure of the A1AT-PPE complex (PDB ID 2D26) has shown a tight interface between A1AT and PPE [27]. The four PPE residues, glutamine-glycine-aspartate-serine (residues 192–195), are located on a loop in close proximity to the P2 proline of A1AT with aspartate 194 providing a negative charge [27]. In the free PPE, the hydroxyl group of serine 195 is hydrogen bonded to the N-ε_2_ of histidine 57 [28]. The structural model of the P357D-PPE complex (Fig 5a) indicates that the carboxyl group of aspartate 357 of P357D can form a hydrogen bond with the hydroxyl group of serine 195 of PPE, which may interfere with the deprotonation of serine 195. The structural model also indicates that like-charge interactions of aspartate 357 of P357D and aspartate 194 of PPE may also interfere with the tight binding of A1AT and PPE (Fig 5a). The serine protease action of PPE is affected in both cases, resulting in conformational changes in the reactive center loop and subsequent cleavages at either the P9 alanine or the P3 isoleucine residue. Other serpins have hydrophobic amino acids (leucine and phenylalanine) or a basic amino acid (arginine) for the P2 residue (Fig 1b), but no acidic amino acids (aspartate and glutamate) were found (data not shown). However, the positive charge at the P2 residue as shown in P357K did not promote A1AT-PPE complex formation.

Although PPE and HNE share structural similarity of the active site (43% amino acid sequence identity), they bind to serpins in a different manner [29] and exhibit different substrate specificity [30, 31]. PPE cleaves substrates at the amino side of alanine, which is relatively small, whereas HNE cleaves substrates at bulkier side chains, such as valine [30]. This supports the determination of cleavage sites in P357D after the P9 alanine and the P3 isoleucine residues by PPE [30]. The surface charge spread over the binding sites of PPE and HNE also supports the difference in their substrate binding (Fig 5b and 5c). These differences may explain why P357L, P357N, and P357W mutants served as better inhibitors of HNE than of PPE (Fig 2a and Table 1).

In conclusion, the size and charge at the P2 residue of A1AT play an important role in interactions with cognate proteases. The negative charge at the P2 residue of A1AT is disfavored for interaction with PPE. The identification of two cleavage sites in P357D using mass spectrometry analysis provides novel insight into the interactions of serpin and proteases.
